# Assessing Severity in Anorexia Nervosa Using Alternative Criteria to the DSM‐5 in a Cross‐Sectional Study

**DOI:** 10.1002/eat.24542

**Published:** 2025-09-10

**Authors:** Esther Henriet, Joanna Norton, Maude Seneque, Laurent Maimoun, Philippe Courtet, Sébastien Guillaume

**Affiliations:** ^1^ Department of Emergency Psychiatry and Acute Care Lapeyronie Hospital Montpellier France; ^2^ University of Montpellier, Institute for Neurosciences of Montpellier (INM), INSERM Montpellier France; ^3^ Institute of Functional Genomics, CNRS, INSERM, University of Montpellier Montpellier France; ^4^ Département of Nuclear Medicine Hôpital Lapeyronie, CHU de Montpellier Montpellier France; ^5^ PhyMedExp, Université de Montpellier, INSERM, CNRS Montpellier France

**Keywords:** anorexia nervosa, body mass index, drive for thinness, DSM‐5, overvaluation of weight and shape, severity

## Abstract

**Objective:**

This study compared overvaluation of weight and shape (OWS), drive for thinness (DT), and their combination (OWS‐DT) as alternative severity classifications for anorexia nervosa (AN) to the fifth edition of the Diagnostic and Statistical Manual of Mental Disorders (DSM‐5) severity classification.

**Method:**

312 AN treatment‐seeking outpatients (mean age = 26.8, SD = 10.2, mean body mass index = 17.3, SD = 2.4) were classified using BMI‐based DSM‐5 criteria (mild/moderate/severe/extreme), OWS (no/yes), DT (no/yes), and OWS‐DT combination (neither/one or the other/both). These classifications were evaluated based on associations with clinical and functional severity indicators, including psychiatric comorbidities, psychopathology via the Eating Disorder Examination Questionnaire and the Eating Disorder Inventory, functional impairment via the Eating Disorders Quality‐of‐Life Questionnaire and Work‐and‐Social Adjustment Scale, and biological markers.

**Results:**

OWS and DT separately were strongly and positively associated with psychiatric comorbidities, psychopathology, and functional impairment. Severity increased across OWS–DT groups, distinguishing mild (neither), moderate (mainly OWS), and severe (both OWS and DT). DT rarely appeared without OWS. DSM‐5 classification alone had limited clinical relevance, primarily reflecting restriction of energy intake. No significant differences emerged across severity classifications for biological markers.

**Discussion:**

Findings offer limited support for DSM‐5 severity criteria alone. OWS and DT showed clearer clinical utility, with the OWS‐DT combination more effectively identifying severity. A stepwise model—screening for OWS followed by DT assessment—may best capture severity in clinical settings.

**Summary**

- This study evaluated emerging alternative severity criteria for anorexia nervosa –overvaluation of weight and shape (OWS) and drive for thinness (DT)‐ compared to the DSM‐5 BMI‐based classification, across clinical dimensions including psychiatric comorbidities, eating disorder psychopathology, physical impact, and functional impairment.
- OWS and DT demonstrated greater clinical utility than the DSM‐5 severity specifier in identifying patients with more severe clinical profiles.
- The combined OWS‐DT criterion showed the strongest discriminative capacity, outperforming each criterion individually.
- As DT rarely occurs without OWS, findings support a stepwise screening approach: assess OWS and evaluate DT only if OWS is present.

## 1 Introduction

Anorexia nervosa (AN) is a severe mental illness affecting between 0.3% and 0.6% of the population (Treasure et al. 2015), with among the highest mortality rates among psychiatric disorders (Zipfel et al. 2015). In addition to life‐threatening physical complications (e.g., multiple organ failure, osteoporosis), AN is associated with high psychiatric comorbidity, including mood and anxiety disorders and suicidality (Zipfel et al. 2015). Its impact on quality of life is substantial, often surpassing that of other psychiatric conditions (Sy et al. 2013).

In 2013, the fifth edition of the Diagnostic and Statistical Manual of Mental Disorders (DSM‐5) introduced a severity classification for AN in adults based on body mass index (BMI) with four categories: mild (BMI ≥ 17 kg/m^2^), moderate (BMI 16–16.99 kg/m^2^), severe (BMI 15–15.99 kg/m^2^), and extreme (BMI < 15 kg/m^2^). These categories were adapted from the World Health Organization guidelines for thinness and may be adjusted by clinicians to reflect clinical symptoms, degree of functional impairment, or need for supervision (American Psychiatric Association and American Psychiatric Association 2013). However, the clinical relevance and predictive validity of this system remain debated. Several studies report weak or no associations between DSM‐5 severity categories and key clinical indicators, including psychopathology, comorbidities, or treatment outcomes (Nakai et al. 2017; Machado et al. 2017; Gianini et al. 2017; Dalle Grave et al. 2018; Krug et al. 2021; Dang et al. 2022, 2023, 2025).

In response, alternative criteria for AN severity have been proposed, notably overvaluation of weight and shape (OWS) and drive for thinness (DT). Both criteria have shown stronger associations with psychopathology, comorbidities, and functional impairment than BMI‐based severity alone (Dang et al. 2021; Gianini et al. 2017; Krug et al. 2021; Mora‐Maltas et al. 2023). However, no studies to date have directly compared them or examined the clinical value of their combination as a composite severity model. Moreover, few investigations have incorporated biological markers of AN severity (Dang et al. 2023).

This cross‐sectional study aimed to compare DSM‐5 severity classification with three alternative approaches: OWS alone, DT alone, and a combined OWS‐DT model with three levels of severity. We assessed their associations with psychiatric comorbidities, eating disorder psychopathology, functional impairment, and physical markers of illness severity to evaluate their clinical utility in capturing AN severity.

## 2 Method

### 2.1 Participants

Patients included in this cross‐sectional study were drawn from two consecutive cohorts of outpatients from the multidisciplinary eating disorders unit at Montpellier University Hospital, France. The first cohort was recruited between February 2012 and October 2014, and the second between May 2017 and January 2020.

Inclusion criteria common to both cohorts were: (1) age between 15 and 45 years, (2) native or fluent in French, and (3) ability to provide informed consent. Exclusion criteria were: (1) inability to understand and complete the clinical evaluation, (2) pregnancy or breastfeeding, and (3) deprivation of liberty by judicial or administrative decision. Informed consent was obtained from all participants and, for those under the age of 18, from official caregivers.

Patients diagnosed with AN as defined by the DSM‐5, with no missing values for the AN severity study variables, were selected. The final sample consisted of 312 patients, 149 from the first cohort and 163 from the second cohort. See flow chart in Supporting Information for more details.

Both studies were approved by ethics committees, CPP Sud‐Mediterranée IV (reference number 11–04‐SC) and CPP Sud‐Est 6 (reference number AU13‐13) for the first and second cohorts, respectively. The study was performed in compliance with the World Medical Association Declaration of Helsinki.

### 2.2 Assessments

In both cohorts, a multidisciplinary clinical assessment was carried out during a full day at the outpatient unit by experienced mental health professionals.

During this visit, patients provided basic demographic information. Self‐reported clinical data were also collected, including age of onset, duration of illness, lowest BMI since illness onset, and history of psychiatric hospitalizations. In accordance with French ethical and legal standard regulations, data on race and ethnicity were not collected in this study.

Eating disorder diagnoses were determined using a best‐estimate procedure, combining clinician assessment based on DSM‐5 criteria, self‐report and clinician‐rated measures, and case discussion in a multidisciplinary clinical team.

Psychopathology was assessed using the Eating Disorder Examination Questionnaire (EDE‐Q) and the Eating Disorder Inventory (EDI‐2) both of which have been validated in French‐speaking populations (Criquillion‐Doublet et al. 1995; Carrard et al. 2011, 2015; Rodgers et al. 2011). Additionally, the cross‐cultural validity of the EDI‐2 has been explored (Podar and Allik 2009).

The EDE‐Q (Fairburn and Beglin 1994) is a 28‐item self‐report questionnaire concerning the past 28 days comprising four subscales—eating restraint, eating concern, weight concern, shape concern—as well as a global score. Higher scores on the EDE‐Q indicate greater levels of eating disorder psychopathology. Internal consistency in our sample was high, with Cronbach's alpha (*α*) equal to 0.84, ranging from 0.80 for eating concern to 0.89 for weight concern.

The EDI‐2 (Garner 1991) is a 91‐item self‐report tool exploring 11 psychological dimensions associated with eating disorders: DT, body dissatisfaction, bulimia, ineffectiveness, perfectionism, interpersonal distrust, interoceptive awareness, maturity fears, asceticism, impulse regulation, and social insecurity. In our sample, internal consistency over the 91 items (*α* = 0.96) was very high, and was greater than 0.82 for all dimensions except for perfectionism (*α* = 0.78), distrust (*α* = 0.77), insecurity (*α* = 0.72), and asceticism (*α* = 0.63).

Psychiatric comorbidities were assessed using the Mini‐International Neuropsychiatric Interview (MINI, French Version 7.0.2) (Sheehan et al. 2016). This structured diagnostic interview, based on DSM‐5 criteria, was conducted by trained professionals.

Functional impairment was evaluated in the second cohort only, using the Eating Disorders Quality of Life questionnaire (EDQOL) with four subscales: Psychological, Physiological, Financial, and work‐related quality of life, and the Work and Social Adjustment Scale (WSAS). Internal consistency was high for EDQOL (*α* = 0.94), its four subscales (*α* ≥ 0.82) and the WSAS (*α* = 0.86).

The EDQOL is a 25‐item self‐report measure of eating disorder‐related quality of life, rated on a 4‐point scale, with higher scores reflecting poorer eating disorder‐related quality of life. It includes four subscales: psychological, physical/cognitive, work/school, and financial. The EDQOL demonstrates high internal consistency and adequate convergent and discriminant validity (Engel et al. 2006).

The WSAS (Mundt et al. 2002) is a 5‐item self‐report scale assessing impairment across work and social domains, with scores ranging from 0 to 8; higher scores indicate greater functional impairment. It is validated for use in populations with eating disorders (Tchanturia et al. 2013).

Physical evaluation included measurements of weight, height, a fasting blood sample, and a metabolic evaluation with indirect calorimetry.

Measured resting energy expenditure (REEm) was performed in all patients between 8:00 a.m. and 8:30 a.m. after overnight fasting and without the practice of physical exercise for 48 h. REEm was assessed by indirect calorimetry (Quark RMR, Cosmed, Rome, Italy), which analyzed oxygen, carbon dioxide, and ventilation in a mixing chamber over a period of at least 30 min. Deviations from normative values were calculated as percentages, categorized into terciles, and dichotomized; the lowest tercile reflected the most pronounced reduction in resting energy expenditure.

Biological anomalies were categorized as follows:

Impaired hepatic assessment was defined by at least one of the following values exceeding lab thresholds: Aspartate Aminotransferase (ASAT) ≥ 64 U/L, Alanine Aminotransferase (ALAT) ≥ 66 U/L, Gamma‐glutamyltransferase (GGT) ≥ 120 U/L, or Alkaline Phosphatase (PAL) ≥ 52.5 U/L.

Biological deficiency was defined by subnormal results on all the following five indicators: phosphate (< 0.8 mmol/L), prealbumin (< 0.2 mg/dL), hemoglobin (< 13 g/dL for men, < 12 g/dL for women), vitamin B12 (< 150 pmol/L), or vitamin D (< 20 ng/mL), reflecting the risk of refeeding syndrome and malnutrition (Proulx‐Cabana et al. 2022).

### 2.3 AN Severity Criteria

In addition to the DSM‐5 severity classification, the following three classifications were created based on alternative criteria:

#### 2.3.1 Overvaluation of Weight and Shape (OWS)

OWS was assessed using two items from the EDE‐Q, “Over the past 4 weeks, has your shape influenced how you feel about (judge, think, evaluate) yourself as a person?” (item 22) and “Over the past 4 weeks has your weight influenced how you feel about (judge, think, evaluate) yourself as a person?” (item 23). Each is rated on a 7‐point scale (0 = not important to 6 = extremely important). Following prior studies, a score ≥ 4 on either item was used to indicate the presence of OWS, classifying participants into OWS‐present or OWS‐absent groups (Gianini et al. 2017; Dang et al. 2023; Grilo et al. 2015).

#### 2.3.2 Drive for Thinness (DT)

DT was measured using the 7‐item DT subscale of the EDI‐2, which captures concerns about dieting, preoccupation with weight, and fear of weight gain (Q1 (inversed), Q7, Q11, Q16, Q25, Q32, Q49). A total score > 14, based on the cut‐off recommended for screening purposes (Garner et al. 1984) and used in recent studies (Krug et al. 2021; Mora‐Maltas et al. 2023; Peñas‐Lledó et al. 2009), indicated the presence of DT.

#### 2.3.3 Combined OWS‐DT Severity Classification

A third model was created by combining OWS and DT. This yielded three levels of severity: neither OWS nor DT, either OWS or DT, and both OWS and DT.

### 2.4 Statistical Analysis

Descriptive statistics were computed for the total sample and according to each of the four severity classifications, using percentages for categorical variables and means with standard deviations (SD) for continuous variables. For each of the four severity classifications, comparisons between response categories were made using the Chi‐squared test for categorical variables (e.g., past hospitalization in psychiatry, current major depressive disorder) and analysis of variance for continuous variables (e.g., age of AN onset, illness duration, BMI, and EDE‐Q and EDI‐2 scores). For analyses involving the OWS and OWS‐DT classifications, EDE‐Q items 22 and 23 were excluded from EDE‐Q Weight Concern and Shape Concern subscales to avoid circularity. A significance threshold of *p* < 0.05 was applied. Significant *p*‐values were corrected for multiple testing between the four severity classifications using the False Discovery Rate procedure (FDR).

The discriminative capacity of the severity measures was based on both the significance level of the association and its effect size. For comparisons with categorical variables, Cramer's V (V) coefficient was given with thresholds varying according to the number of degrees of freedom (see Tables for thresholds and interpretation) (Cohen 1988). For continuous variables, partial eta‐squared coefficients (*η*) were calculated with values ≥ 0.01 and < 0.06 considered as small, ≥ 0.06 and < 0.14 as medium, and ≥ 0.14 as large (Richardson 2011). Effect sizes were presented with their 95% Confidence Intervals (95% CI) (see Supporting Information). We also examined whether the associations between the three alternative severity classifications (OWS, DT, OWS‐DT) and selected socio‐demographic (age), clinical (age at AN onset, illness duration, comorbidities), and functional variables were moderated by DSM‐5 severity (BMI). None of the tested interactions reached statistical significance. Statistical analyses were performed using SAS Enterprise Guide 7.15 (SAS Institute Inc. Cary, North Carolina).

## 3 Results

### 3.1 Sample Description and AN Severity Classifications

Of the 312 patients included, 94.2% (*n* = 294) were female, with a mean age of 26.8 years (SD = 10.2). Most participants were single (70.7%); 49.7% were students and 32.1% were employed. Regarding educational level, 35.3% had completed secondary education and 18% had attained a university degree (data not shown).

Based on DSM‐5 severity definition, 161 (51.6%) patients were categorized as mild, 56 (17.9%) as moderate, 48 (15.4%) as severe, and 47 (15.1%) as extreme (Table 1). For the two alternative classifications, 211 (67.6%) patients reached criteria for OWS and 113 (36.2%) for DT. As ordinal variables, OWS (median [range]: 8 [0–12]) and DT (median [range]: 12 [0–21] for DT) were strongly correlated (Spearman correlation coefficient *r* = 0.60, *p* < 0.0001). The binary severity classifications were also strongly associated (*Χ* ^2^ = 44.8, *p* < 0.0001, Cramer’ V coefficient = 0.38). Regarding their combinations, 91 patients (29.2%) had neither OWS nor DT, while 118 patients (37.8%) exhibited either one or the other. Among these, the vast majority—108 patients (91.5%)—had OWS only, whereas DT without OWS was rare (*n* = 10, 8.5%). Finally, 103 patients (33%) met the criteria for both OWS and DT. DSM‐5 severity did not differ significantly according to OWS, DT, or OWS‐DT, and there was no interaction between DT and OWS in association with DSM‐5 severity. No significant differences in sociodemographic variables were observed across the four severity classifications.

**TABLE 1:** Socio‐demographic characteristics of the patients according to severity classification (*N* = 312).

Severity classification	DSM‐5 severity level				OWS		DT		OWS‐DT		
	Mild	Moderate	Severe	Extreme	No	Yes	No	Yes	Neither	OWS or DT	OWS + DT
	% (*n*)	% (*n*)	% (*n*)	% (*n*)	% (*n*)	% (*n*)	% (*n*)	% (*n*)	% (*n*)	% (*n*)	% (*n*)
	51.6 (161)	17.9 (56)	15.4 (48)	15.1 (47)	32.4 (101)	67.6 (211)	63.8 (199)	36.2 (113)	29.2 (91)	37.8 (118)	33.0 (103)
Age (yrs) (mean, SD)	26.8 (10.2)	25.5 (7.1)	25.2 (10.8)	29.9 (11.8)	26.1 (10.1)	27.1 (10.2)	26.6 (9.6)	27.1 (11.1)	25.6 (9.5)	27.8 (10.0)	26.7 (10.8)
Gender											
Male	9.3 (15)	5.4 (3)	0.0 (0)	0.0 (0)	6.9 (7)	5.2 (11)	7.0 (14)	3.5 (4)	7.7 (7)	5.9 (7)	3.9 (4)
Female	90.7 (146)	94.6 (53)	100.0 (48)	100.0 (47)	93.1 (94)	94.8 (200)	93.0 (185)	96.5 (109)	92.3 (84)	94.1 (111)	96.1 (99)
Marital status											
Single	66.5 (107)	71.4 (40)	79.2 (38)	76.1 (35)	71.3 (72)	70.5 (148)	71.7 (142)	69.0 (78)	73.6 (67)	68.4 (80)	70.9 (73)
Married/living together	28.0 (45)	21.4 (12)	14.6 (7)	17.4 (8)	20.8 (21)	24.3 (51)	21.7 (43)	25.7 (29)	20.9 (19)	22.2 (26)	26.2 (27)
Divorced/separated	5.6 (9)	7.1 (4)	6.3 (3)	6.5 (3)	7.9 (8)	5.2 (11)	6.6 (13)	5.3 (6)	5.5 (5)	9.4 (11)	2.9 (3)
Occupation											
Employed	33.5 (54)	26.8 (15)	29.2 (14)	36.2 (17)	33.7 (34)	31.3 (66)	34.2 (68)	28.3 (32)	33.0 (30)	35.6 (42)	27.2 (28)
Unemployed	11.2 (18)	16.1 (9)	12.5 (6)	12.8 (6)	8.9 (9)	14.2 (30)	12.1 (24)	13.3 (15)	8.8 (8)	14.4 (17)	13.6 (14)
Student/training	50.3 (81)	51.8 (29)	52.1 (25)	42.6 (20)	52.5 (53)	48.3 (102)	47.2 (94)	54.0 (61)	53.9 (49)	41.5 (49)	55.3 (57)
Illness, invalidity	5.0 (8)	5.4 (3)	6.3 (3)	8.5 (4)	5.0 (5)	6.2 (13)	6.5 (13)	4.4 (5)	4.4 (4)	8.5 (10)	3.9 (4)
Level of education^a^											
Primary or less	42.6 (63)	52.8 (28)	48.9 (22)	51.2 (22)	51.7 (47)	44.4 (88)	43.3 (78)	52.3 (57)	49.4 (40)	41.3 (45)	50.5 (50)
Secondary	39.2 (58)	22.6 (12)	37.8 (17)	34.9 (15)	31.9 (29)	36.9 (73)	38.3 (69)	30.3 (33)	34.6 (28)	38.5 (42)	32.3 (32)
University	18.2 (27)	24.5 (13)	13.3 (6)	14.0 (6)	16.5 (15)	18.7 (37)	18.3 (33)	17.4 (19)	16.1 (13)	20.2 (22)	17.2 (17)

*Note*: In accordance with French ethical and legal standard regulations, data on race and ethnicity were not collected in this study.

Abbreviations: DT, drive for thinness; OWS, overvaluation of weight and shape; SD, standard deviation.

^a^ 7% missing values for level of education.

### 3.2 Anorexia Nervosa Subtype Distribution and Clinical Characteristics

Regarding type of AN, 54.4% were classified as having the restricting subtype and 45.6% as having the binge‐eating/purging subtype. DSM‐5 severity level was associated with type of AN, with a higher percentage of restricting subtype in the moderate (66.1%) to extreme (71.7%) severity levels compared to the mild level (42.1%), with a medium effect size (V = 0.26, 95% CI: 0.16–0.37) (Table 2; Table S1). The opposite was observed for the three alternative classifications; the proportion of restricting subtype decreased across increasing severity levels. For OWS‐DT, the restricting subtype decreased from 69.2% for those with neither OWS nor DT to 53.9% for those with mainly OWS to 41.6% for those with both, with a medium effect size (V = 0.22) despite a large 95% CI ranging from a small (V = 0.11) to medium (V = 0.32) effect size.

**TABLE 2:** History and psychopathology of anorexia nervosa according to severity classification (*N* = 312)^a^.

Severity classification	DSM‐5 severity level					OWS			DT			OWS‐DT			
	Mild	Moderate	Severe	Extreme	V/p	No	Yes	V/p	No	Yes	V/p	Neither	OWS or DT	OWS + DT	V/p
Subtype of AN %, (*n*)^b^															
Restricting	42.1 (67)	66.1 (37)	64.6 (31)	71.7 (33)	**0.26**	67.3 (68)	48.1 (100)	0.18	61.1 (121)	42.3 (47)	0.18	69.2 (63)	53.9 (63)	41.6 (42)	**0.22**
Binge‐eating/purging	57.9 (92)	33.9 (19)	35.4 (17)	28.3 (13)	0.0004	32.7 (33)	51.9 (108)	0.001	38.9 (77)	57.7 (64)	0.002	30.8 (28)	46.1 (54)	58.4 (59)	0.001

	Mean (SD)	Mean (SD)	Mean (SD)	Mean (SD)	*η* ^2^/*p*	Mean (SD)	Mean (SD)	*η* ^2^/*p*	Mean (SD)	Mean (SD)	*η* ^2^/*p*	Mean (SD)	Mean (SD)	Mean (SD)	*η* ^2^/*p*
Onset of AN (yrs)	21.1 (9.4)	20.2 (6.0)	20.6 (8.5)	20.7 (8.2)	0.002	20.4 (7.9)	21.0 (8.9)	0.0009	21.0 (8.1)	20.4 (9.4)	0.0009	20.5 (8.0)	21.2 (8)	20.5 (9.7)	0.002
					0.94			0.64			0.64				0.82
Illness duration (yrs)	5.2 (6.3)	5.5 (6.7)	5.1 (9.6)	8.1 (9.7)	0.02	5.1 (7.9)	5.8 (7.2)	0.002	5.2 (6.9)	6.4 (8.3)	0.007	4.5 (6.3)	6.1 (8.2)	5.9 (7.3)	0.009
					0.2			0.5			0.22				0.32
BMI at assessment	19.0 (2.1)	16.6 (0.3)	15.5 (0.3)	14.0 (0.9)	—	16.7 (2.0)	17.5 (2.6)	0.02	16.9 (2.0)	17.9 (2.9)	0.04	16.7 (2.0)	17.1 (2.0)	18.0 (3.0)	0.05
								0.007			0.0009				0.0009
Lowest BMI	16.0 (2.1)	14.8 (1.7)	14.6 (1.0)	13.1 (1.5)	**0.23**	14.9 (1.8)	15.2 (2.2)	0.004	15 (2.0)	15.3 (2.2)	0.004	14.9 (1.9)	15.2 (2.1)	15.3 (2.3)	0.006
					0.0004			0.37			0.37				0.39
6‐month change in BMI^c^	0.3 (1.7)	−0.9 (2.3)	−0.8 (1.5)	−1.4 (1.9)	**0.12**	−0.5 (1.9)	−0.3 (2.0)	0.003	−0.4 (1.8)	−0.3 (2.3)	0.0001	−0.4 (1.8)	−0.4 (1.9)	−0.3 (2.2)	0.001
					0.0004			0.82			0.87				0.87
EDE‐Q scores															
Restraint	2.9 (1.9)	3.0 (2.0)	2.8 (2.2)	2.9 (2.0)	0.002	1.8 (1.8)	3.5 (1.8)	**0.16**	2.1 (1.8)	4.4 (1.2)	**0.33**	1.5 (1.5)	2.8 (1.9)	4.4 (1.2)	**0.35**
					0.92			0.0001			0.0001				0.0001
Eating concern	2.7 (1.7)	2.6 (1.7)	2.4 (1.6)	2.5 (1.7)	0.004	1.4 (1.4)	3.2 (1.5)	**0.25**	1.9 (1.6)	3.9 (1.1)	**0.31**	1.2 (1.3)	2.6 (1.5)	3.9 (1.1)	**0.40**
					0.72			0.0001			0.0001				0.0001
Weight concern	3.5 (1.8)	3.2 (1.7)	3.1 (1.7)	3.0 (1.8)	0.014	1.8 (1.5)	3.9 (1.5)	**0.31**	2.5 (1.5)	4.8 (1.0)	**0.4**	1.6 (1.3)	3.2 (1.4)	4.8 (1.0)	**0.51**
					0.22			0.0001			0.0001				0.0001
Shape concern	4.0 (1.7)	3.7 (1.7)	3.6 (1.6)	3.4 (1.7)	0.02	2.3 (1.6)	4.5 (1.3)	**0.35**	3.0 (1.6)	5.2 (0.8)	**0.36**	2.1 (1.3)	3.9 (1.4)	5.2 (0.8)	**0.52**
					0.13			0.0001			0.0001				0.0001
EDI‐2 scores															
Drive for thinness	12.1 (6.1)	9.9 (6.9)	9.5 (6.5)	10.1 (6.2)	0.03	6.6 (5.6)	13.1 (5.7)	0.22	7.1 (4.6)	17.8 (1.8)	—	5.4 (4.4)	9.3 (4.9)	17.8 (1.7)	—
					0.02			0.0002							
Bulimia	5.1 (5.6)	3.5 (5.4)	2.6 (4.4)	2.0 (3.8)	**0.06**	1.9 (3.4)	5.0 (5.7)	**0.07**	2.6 (4.1)	6.5 (6.2)	**0.13**	1.7 (3.3)	3.3 (4.5)	6.7 (6.3)	**0.15**
					0.0004			0.0001			0.0001				0.0001
Body satisfaction	13.5 (7.2)	11.5 (7.0)	10.6 (5.7)	10.4 (4.6)	0.04	8.6 (5.0)	14.0 (6.7)	**0.14**	9.4 (5.5)	17.3 (5.6)	**0.32**	7.8 (4.4)	11.2 (6.0)	17.4 (5.7)	**0.34**
					0.005			0.0001			0.0001				0.0001
Ineffectiveness	11.4 (7.5)	10.6 (7.8)	10.5 (7.3)	10.7 (7.3)	0.003	6.8 (6.0)	13.0 (7.2)	**0.15**	8.5 (6.5)	15.4 (7.0)	**0.2**	6.0 (5.2)	11.1 (6.8)	15.5 (7.0)	**0.25**
					0.82			0.0001			0.0001				0.0001
Perfectionism	7.7 (4.9)	6.9 (4.6)	8.2 (4.8)	6.4 (4.1)	0.02	5.8 (4.2)	8.2 (4.8)	**0.06**	6.4 (4.5)	9.3 (4.5)	**0.09**	5.4 (4.1)	7.4 (4.6)	9.3 (4.7)	**0.10**
					0.19			0.0001			0.0001				0.0001
Interpersonal distrust	6.1 (4.4)	6.0 (4.5)	6.5 (4.6)	5.9 (3.9)	0.002	5.1 (3.7)	6.6 (4.6)	0.03	5.7 (4.3)	6.8 (4.4)	0.01	4.9 (3.6)	6.5 (4.7)	6.7 (4.5)	0.03
					0.91			0.0001			0.07				0.008
Interoceptive awareness	11.2 (7.3)	10.8 (8.6)	8.9 (7.1)	10.0 (7.0)	0.01	6.2 (5.9)	12.7 (7.2)	**0.17**	7.9 (6.4)	15.4 (6.7)	**0.24**	5.2 (5.0)	10.7 (6.7)	15.4 (6.9)	**0.29**
					0.27			0.0002			0.0002				0.0001
Maturity fears	6.2 (5.3)	7.5 (6.1)	6.7 (5.7)	6.5 (5.6)	0.007	4.9 (4.5)	7.3 (5.8)	0.04	5.5 (4.7)	8.4 (6.3)	**0.07**	4.6 (4.3)	6.3 (5.1)	8.5 (6.4)	**0.08**
					0.5			0.0001			0.0001				0.0001
Asceticism	7.9 (4.7)	6.9 (4.6)	7.1 (4.3)	6.9 (4.3)	0.01	5.2 (3.6)	8.6 (4.5)	**0.12**	5.8 (3.8)	10.4 (4.4)	**0.23**	4.6 (3.0)	7.2 (4.2)	10.3 (4.4)	0.25
					0.33			0.0001			0.0001				0.0001
Impulse regulation	7.0 (6.4)	6.1 (5.6)	6.0 (6.9)	6.0 (6.7)	0.005	3.5 (5.0)	8.0 (6.5)	**0.11**	4.9 (5.7)	9.5 (6.5)	**0.12**	3.1 (4.9)	6.5 (5.7)	9.7 (6.7)	**0.17**
					0.65			0.0001			0.0001				0.0001
Social insecurity	7.9 (4.8)	6.9 (5.1)	7.8 (4.8)	7.9 (5.1)	0.006	5.5 (4.5)	8.8 (4.7)	**0.1**	6.6 (4.7)	9.8 (4.6)	**0.1**	4.9 (4.2)	8.3 (4.6)	9.6 (4.7)	**0.15**
					0.61			0.0001			0.0001				0.0001

*Note*: Medium to large effect sizes based on Cramer's V with the following thresholds are given in bold: for DSM‐5 severity level (3 df): [0.06–0.17[: small, [0.17–0.29[: medium, [0.29 +: large; for OWS and DT (1 df): [0.10–0.30[: small, [0.30–0.50[: medium, [0.50 +: large; for OWS‐DT (2 df): [0.07–0.21[: small, [0.21–0.35[: medium, [0.35 +: large.

Abbreviations: AN, anorexia nervosa; BMI, body mass index expressed in kg/m^2^; DT, drive for thinness; EDE‐Q, eating disorder examination questionnaire; EDI‐2, eating disorder inventory; *η* ^2^, partial eta‐squared coefficients effect size for continuous variables; OWS, overvaluation of weight and shape; SD, standard deviation.

^a^ Missing values: for EDE‐Q and EDI‐2 (≤ 1.5%); BMI at assessment (6%); duration of illness, lowest BMI, and change in BMI (18%–20%).

^b^ Missing values for three patients.

^c^ Difference between BMI at 6 months prior to assessment and BMI at assessment.

Patients with binge‐eating/purging subtype AN also differed significantly from the restricting subtype across multiple clinical indicators. They had higher educational levels, a higher prevalence of suicide attempts, current MDD, and anxiety disorders, as well as a longer illness duration and a higher BMI. Psychopathology scores were significantly higher across all EDI‐2 and EDE‐Q dimensions (except Interpersonal Distrust and Maturity Fears). On functional measures, only the EDQOL psychological and financial domains were significantly worse in the binge‐eating/purging group.

### 3.3 Characteristics of AN: History and Psychopathology

As expected, when considering the lowest BMI measured since illness onset, the mean value decreased as DSM‐5 severity level increased, with a large effect size (*η* ^2^ = 0.23, 95% CI: 0.16–0.34). Likewise, the average negative change in BMI at assessment compared to 6 months earlier increased with DSM‐5 severity. For the three alternative classifications, BMI at assessment was significantly higher in the event of OWS, DT, and the co‐occurrence of the two, although effect sizes were small.

Regarding eating disorder psychopathology, the clinical dimensions assessed by the EDE‐Q and the EDI‐2 generally did not vary across DSM‐5 severity levels, except for drive to thinness, bulimia, and body satisfaction. Scores decreased as severity increased, with a medium effect size for bulimia only (*η* ^2^ = 0.06, 95% CI: 0.02–0.11). Patients classified with OWS or DT showed significantly higher scores across most clinical dimensions (except interpersonal distrust and maturity fears), with medium to large effect sizes. Among the three alternative classifications, the combined OWS‐DT model demonstrated the strongest and most consistent associations with psychopathology scores, showing a graded pattern across severity levels.

### 3.4 Psychiatric Comorbidities

Table 3 shows the distribution of psychiatric comorbidities according to the different AN severity classifications. Although DSM‐5 severity levels did not follow a linear pattern in relation to suicide attempts or past major depressive disorder (MDD), the mild severity group showed the highest prevalence for both. These differences between DSM‐5 severity categories were statistically meaningful; effect sizes were medium with large confidence intervals (Table S2). The opposite was observed for OWS, DT, and their combination, with the frequency of suicide attempts increasing hand‐in‐hand with the severity levels of OWS‐DT, reaching 30.1% for patients with both OWS and DT, despite small effect sizes. The frequency of current and past MDD was also higher in the event of OWS and DT. Current MDD increased from 16.5% among patients with neither OWS nor DT, to 28.8% among those with mainly OWS, and reached 45.4% among those with both OWS and DT, with a medium effect size (V = 0.25, 95% CI: 0.15–0.36). The same pattern was observed for anxiety disorders. Other comorbidities including bipolar disorder, post‐traumatic stress disorder, and substance use disorder were examined; however, comparisons were precluded due to insufficient sample sizes.

**TABLE 3:** Prevalence of psychiatric comorbidities across anorexia nervosa severity classifications (*N* = 312)^a^.

	DSM‐5 severity level					OWS			DT			OWS‐DT			
	Mild	Moderate	Severe	Extreme		No	Yes		No	Yes		Neither	OWS or DT	OWS + DT	
	% (*n*)	% (*n*)	% (*n*)	% (*n*)	V/p	% (*n*)	% (*n*)	V/p	% (*n*)	% (*n*)	V/p	% (*n*)	% (*n*)	% (*n*)	V/p
Past hospitalization in psychiatry															
No	55.9 (90)	57.1 (32)	70.8 (34)	54.3 (25)	0.11	65.3 (66)	54.8 (115)	0.10	64.3 (128)	47.3 (53)	0.17	68.1 (62)	59.3 (70)	48.0 (49)	0.16
Yes	44.1 (71)	42.9 (24)	29.2 (14)	45.7 (21)	0.28	34.7 (35)	45.2 (95)	0.11	35.7 (71)	52.7 (59)	0.02	31.9 (29)	40.7 (48)	52.0 (53)	0.04
Suicide attempt															
No	73.8 (118)	87.5 (49)	79.2 (38)	95.7 (45)	**0.21**	88.0 (88)	76.8 (162)	0.13	86.4 (171)	69.9 (79)	0.20	90.0 (81)	82.2 (97)	69.9 (72)	0.20
Yes	26.3 (42)	12.5 (7)	20.8 (10)	4.3 (2)	0.005	12.0 (12)	23.2 (49)	0.02	13.6 (27)	30.1 (34)	0.002	10.0 (9)	17.8 (21)	30.1 (31)	0.004
Current MDD															
No	66.0 (99)	72.7 (40)	71.7 (33)	72.7 (32)	0.07	81.7 (76)	63.4 (128)	0.18	77.1 (145)	55.1 (59)	0.23	83.5 (71)	71.2 (79)	54.6 (54)	**0.25**
Yes	34.0 (51)	27.3 (15)	28.3 (13)	27.3 (12)	0.7	18.3 (17)	36.6 (74)	0.003	22.9 (43)	44.9 (48)	0.0002	16.5 (14)	28.8 (32)	45.4 (45)	0.0002
Past MDD															
No	39.3 (59)	44.4 (24)	60.9 (28)	56.8 (25)	**0.17**	61.3 (57)	39.3 (79)	0.21	49.5 (93)	40.6 (43)	0.09	62.3 (53)	39.6 (44)	39.8 (39)	**0.21**
Yes	60.7 (91)	55.6 (30)	39.1 (18)	43.2 (19)	0.04	38.7 (36)	60.7 (122)	0.002	50.5 (95)	59.4 (63)	0.14	37.7 (32)	60.4 (67)	60.2 (59)	0.004
Anxiety disorders															
No	52.7 (79)	58.5 (31)	46.7 (21)	47.7 (21)	0.08	72.8 (67)	42.5 (85)	0.28	58.1 (108)	41.5 (44)	0.16	75.0 (63)	44.6 (49)	40.8 (40)	**0.29**
Yes	47.3 (71)	41.5 (22)	53.3 (24)	52.3 (23)	0.62	27.2 (25)	57.5 (115)	0.0002	41.9 (78)	58.5 (62)	0.009	25.0 (21)	55.4 (61)	59.2 (58)	0.0002
OCD															
No	89.3 (134)	88.7 (47)	82.2 (37)	84.1 (37)	0.09	93.5 (86)	84.5 (169)	0.13	89.8 (168)	82.9 (87)	0.10	92.9 (78)	88.3 (98)	81.4 (79)	0.14
Yes	10.7 (16)	11.3 (6)	17.8 (8)	15.9 (7)	0.55	6.5 (6)	15.5 (31)	0.12	10.2 (19)	17.1 (18)	0.12	7.1 (6)	11.7 (13)	18.6 (18)	0.12

*Note*: Medium to large effect sizes based on Cramer's V with the following thresholds are given in bold: for DSM‐5 severity level (3 df): [0.06–0.17[: small, [0.17–0.29[: medium, [0.29 +: large; for OWS and DT (1 df): [0.10–0.30[: small, [0.30–0.50[: medium, [0.50 +: large; for OWS‐DT (2 df): [0.07–0.21[: small, [0.21–0.35[: medium, [0.35 +: large.

Abbreviations: DT, drive for thinness; MDD, major depressive disorder; OCD, obsessive compulsive disorder; OWS, overvaluation of weight and shape; V, Cramer's V effect size for categorial variables.

^a^ Missing values: ≤ 3% for current MDD, past MDD, anxiety disorders, OCD.

### 3.5 Biological and Metabolic Markers

Resting energy expenditure decreased significantly with increasing DSM‐5 severity, with a large effect size (V = 0.35, 95% CI: 0.24–0.45). Although not statistically significant, there was a trend toward greater biological abnormalities—including nutrient deficiencies and hepatic function impairments—among patients with DT and those with both OWS and DT; however, these associations showed small effect sizes (Table 4).

**TABLE 4:** Biological and metabolic markers according to severity classification (*N* = 312)^a^.

Severity classification	DSM‐5 severity level					OWS			DT			OWS‐DT			
	Mild	Moderate	Severe	Extreme		No	Yes		No	Yes		Neither	OWS or DT	OWS + DT	
	% (*n*)	% (*n*)	% (*n*)	% (*n*)	V/p	% (*n*)	% (*n*)	V/p	% (*n*)	% (*n*)	V/p	% (*n*)	% (*n*)	% (*n*)	V/p
Resting energy expenditure (terciles)															
≥ Medium (T1&T2)	80.7 (125)	64.2 (34)	43.5 (20)	42.9 (18)	**0.35**	60.2 (56)	69.5 (141)	0.09	66.8 (127)	66.0 (70)	0.008	61.6 (53)	69.4 (77)	67.7 (67)	0.07
Low (T3)	19.4 (30)	35.9 (19)	56.5 (26)	57.1 (24)	< 0.0001	39.8 (37)	30.5 (62)	0.24	33.2 (63)	34.0 (36)	0.89	38.4 (33)	30.6 (34)	32.3 (32)	0.67
Hepatic assessment															
Normal	47.7 (72)	41.5 (22)	58.1 (25)	51.1 (23)	0.10	50.6 (46)	47.8 (96)	0.03	52.5 (96)	42.2 (46)	0.10	48.8 (40)	56.4 (62)	40.0 (40)	0.14
Impaired	53.3 (79)	58.5 (31)	41.9 (18)	48.9 (22)	0.56	49.5 (45)	52.2 (105)	0.66	47.5 (87)	57.8 (63)	0.18	51.2 (42)	43.6 (48)	60.0 (60)	0.18
Deficiency in all five biological tests															
No	74.5 (108)	79.6 (35)	88.6 (39)	65.6 (21)	0.15	80.0 (64)	75.1 (139)	0.05	79.9 (135)	70.8 (68)	0.10	78.9 (56)	81.3 (87)	69.0 (60)	0.13
Yes	25.5 (37)	20.5 (9)	11.4 (5)	34.4 (11)	0.15	20.0 (16)	24.9 (46)	0.39	20.1 (34)	29.2 (28)	0.15	21.1 (15)	18.7 (20)	31.0 (27)	0.15
Biological deficiency and hepatic impairment															
No	85.5 (124)	88.6 (39)	90.9 (40)	81.3 (26)	0.08	90.0 (72)	84.9 (157)	0.07	89.9 (152)	80.2 (77)	0.14	88.7 (63)	91.6 (98)	78.2 (68)	0.17
Yes	14.5 (21)	11.4 (5)	9.1 (4)	18.8 (6)	0.62	10.0 (8)	15.1 (28)	0.35	10.1 (17)	19.8 (19)	0.06	11.3 (8)	8.4 (9)	21.8 (19)	0.06

*Note*: Medium to large effect sizes based on Cramer's V with the following thresholds are given in bold: for DSM‐5 severity level (3 df): [0.06–0.17[: small, [0.17–0.29[: medium, [0.29 +: large; for OWS and DT (1 df): [0.10–0.30[: small, [0.30–0.50[: medium, [0.50 +: large; for OWS‐DT (2 df): [0.07–0.21[: small, [021–0.35[: medium, [0.35 +: large.

Abbreviations: DT, drive for thinness; OWS, overvaluation of weight and shape; Resting energy expenditure (terciles), deviation from normative values as percentages; T1 and T2, first two terciles deviating from normative values; T3, lowest tercile as in most pronounced reduction in resting energy expenditure; V, Cramer's V effect size for categorial variables.

^a^ Missing values: ≤ 3% for resting energy expenditure and hepatic assessment; 15% for biological deficiency.

### 3.6 Functional Consequences

No significant differences in functional impairment, as measured by WSAS or EDQOL, were found across DSM‐5 severity levels (Table 5). By contrast, both WSAS and EDQOL scores increased significantly with the presence of OWS, DT and across OWS‐DT severity levels with medium to large effect sizes, despite wide confidence intervals (Table S4). The psychological subscale of the EDQOL demonstrated large effect sizes for all three alternative classifications; the largest was for OWS‐DT (*η* ^2^ = 0.28, 95% CI: 0.19–0.41). The financial domain showed no significant association.

**TABLE 5:** Functional impact according to severity classification (*N* = 163).

Severity classification	DSM‐5 severity level					OWS			DT			OWS‐DT			
	Mild	Moderate	Severe	Extreme		No	Yes		No	Yes		Neither	OWS or DT	OWS + DT	
	Mean (SD)	Mean (SD)	Mean (SD)	Mean (SD)	*η* ^2^/*p*	Mean (SD)	Mean (SD)	*η* ^2^/*p*	Mean (SD)	Mean (SD)	*η* ^2^/*p*	Mean (SD)	Mean (SD)	Mean (SD)	*η* ^2^/*p*
WSAS score	18.2 (10.4)	2.1 (1.0)	22.9 (9.9)	21.1 (10.6)	0.03	15.0 (11.4)	21.3 (9.7)	**0.07**	17.3 (11.0)	22.7 (9.0)	**0.06**	13.4 (10.9)	20.8 (10.2)	22.3 (9.0)	**0.12**
					0.18			0.001			0.001				0.001
EDQOL^a^															
Psychological	2.1 (1.0)	2.4 (1.0)	2.3 (1.0)	2.6 (0.7)	0.03	1.6 (1.0)	2.5 (0.8)	**0.17**	1.9 (1.0)	2.7 (0.6)	**0.21**	1.4 (0.9)	2.3 (0.9)	2.7 (0.6)	**0.28**
					0.17			0.001			0.001				0.001
Physiological	1.8 (1.0)	2.0 (1.0)	2.2 (0.8)	2.1 (0.7)	0.02	1.6 (0.9)	2.1 (0.9)	0.04	1.7 (0.9)	2.2 (0.8)	**0.08**	1.5 (0.9)	2.0 (0.9)	2.2 (0.9)	**0.08**
					0.42			0.01			0.002				0.002
Financial	0.5 (0.7)	0.5 (0.6)	0.5 (0.9)	0.5 (0.9)	0.0005	0.4 (0.7)	0.6 (0.8)	0.02	0.4 (0.7)	0.7 (0.8)	0.03	0.3 (0.7)	0.4 (0.7)	0.7 (0.8)	0.04
					0.99			0.17			0.11				0.14
Work	1.0 (1.1)	1.3 (1.3)	0.9 (0.9)	0.9 (1.2)	0.01	0.7 (0.9)	1.2 (1.2)	0.04	0.8 (1.0)	1.4 (1.2)	**0.07**	0.6 (0.9)	0.9 (1.1)	1.4 (1.2)	**0.08**
					0.68			0.01			0.004				0.004

*Note*: Medium to large effect sizes based on partial eta‐square (*η* ^2^) with the following thresholds are given in bold: 0.01 ≤ *η* ^2^ < 0.06: small; 0.06 ≤ *η* ^2^ < 0.14: medium; *η* ^2^ ≥ 0.14: large.

Abbreviations: DT, drive for thinness; EDQOL, eating disorders quality of life questionnaire; OWS, overvaluation of weight and shape; SD, standard deviation; WSAS, work and social adjustment scale; *η* ^2^, partial eta‐square (η^2^) effect size for continuous variables.

^a^ Missing values for EDQOL, 9% for Work; 11% for financial.

## 4 Discussion

This study evaluated a novel approach to assessing AN severity by combining two alternative criteria recently proposed in the literature: OWS and DT. We examined associations between these classifications and psychiatric comorbidities, eating disorder psychopathology, physical consequences, and functional impairment. Our findings support previous evidence that DSM‐5 severity categories have limited utility, while OWS and DT offer better identification of clinically severe patients. Notably, we demonstrated for the first time that OWS and DT equally proved clinical value to distinguish more severe patients, but DT was rarely in the absence of OWS. The combined criterion OWS‐DT emerged as being more efficient to identify severity in a stepped way than alternative criteria individually.

### 4.1 The DSM‐5 Severity Rating

Our findings provided limited support for the DSM‐5 severity classification as a gold standard to assess severity in AN. Except for an inverse association with past MDD and suicide attempts (more frequent in the mild category, with medium effect sizes), DSM‐5 categories did not differentiate patients in terms of psychiatric comorbidities. These results align with previous studies (Dang et al. 2023; Gianini et al. 2017; Krug et al. 2021). We found no significant differences in eating disorder psychopathology across DSM‐5 severity categories, consistent with earlier reports (Gianini et al. 2017; Krug et al. 2021; Machado et al. 2017), and some studies have even shown greater psychopathology in higher BMI categories (Dang et al. 2023; Nakai et al. 2017).

Functional impairment showed no association with DSM‐5 severity—again consistent with prior findings (Dang et al. 2023; Gianini et al. 2017).

Concerning physical consequences, this study is one of the few to explore associations between measures of the physical impact of AN and DSM‐5 severity. As in the study by (Dang et al. 2023) no significant differences were found with regard to biological status. While calorimetry showed lower resting energy expenditure in the severe and extreme DSM‐5 severity categories, this likely reflects the physiological impact of lower BMI and is in keeping with the findings of a study using body fat percentage as a proxy for BMI (Krug et al. 2021).

### 4.2 Alternative Criteria: Overvaluation of Weight and Shape or Drive for Thinness or Both?

Patients meeting OWS criteria showed higher rates of psychiatric comorbidities, including current and past depression, anxiety disorders, and past suicide attempts, along with increased eating disorder psychopathology and greater functional impairment across several domains of the EDQOL and WSAS. These findings are consistent with previous research (Dang et al. 2023; Gianini et al. 2017) and support the utility of OWS as a clinically relevant indicator of psychiatric burden and reduced quality of life.

Similarly, DT was associated with markers of greater psychopathology and psychiatric severity, including higher rates of current depression, anxiety disorders, psychiatric hospitalization, and past suicide attempts. Functional impairment was also elevated across all EDQOL domains among patients with DT. These results align with studies highlighting DT's ability to capture more severe forms of AN (Krug et al. 2021; Mora‐Maltas et al. 2023).

In our sample, 67.7% of patients met the threshold for OWS, while 36.2% met the criteria for DT. These prevalence rates are in line with those described in previous studies (Dang et al. 2025, 2023; Gianini et al. 2017; Mora‐Maltas et al. 2023). Among those presenting with either OWS or DT, the vast majority (91.5%) had OWS alone, whereas DT without OWS was relatively rare (8.5%). This pattern suggests that OWS functions as a broader, more inclusive severity marker, while DT may identify a subgroup with heightened psychological vulnerability within the OWS‐positive population.

Notably, 29.2% of patients did not meet criteria for either OWS or DT. This subgroup exhibited lower levels of comorbidities, psychopathology, and functional impairment, suggesting overall lower clinical severity. However, the absence of OWS or DT may also reflect limited illness insight or denial of severity, both common features in AN. This group had a significantly lower BMI at assessment, although the effect size was small.

Although OWS and DT were moderately correlated and significantly associated in the categorical forms, the partial dissociation observed, particularly the frequent presence of OWS without DT, suggests that these constructs reflect related but non‐redundant dimensions of AN severity. The moderate strength of the correlation (*r* = 0.60) further supports the idea that OWS and DT should not be considered proxies for one another. The fact that DT was rarely observed in the absence of OWS may point to a clinical sequence or shared underlying mechanism, whereby the OWS precedes or facilitates the emergence of a DT.

Importantly, the combined OWS‐DT demonstrated a stepwise increase in psychiatric comorbidities, eating disorder psychopathology, and functional impairment. Patients with both OWS and DT exhibited the highest levels of clinical severity, with medium to large effect sizes, set however in large confidence intervals. Compared to using OWS or DT alone, the combined criterion offered greater discriminative capacity across nearly all clinical dimensions. These findings suggest that OWS‐DT provides a more robust and multidimensional framework for assessing AN severity.

In addition, exploratory analyses showed meaningful clinical differences between restricting and binge‐eating/purging subtypes. The restricting subtype was more frequent among patients with moderate to extreme DSM‐5 severity, and among those without OWS nor DT. In contrast, the binge‐eating/purging subtype was more common among individuals classified with OWS and DT, particularly in the OWS‐DT group, and was associated with greater clinical severity. These findings suggest that AN subtypes may interact with severity classification and that cognitive‐affective features such as OWS and DT may be more prominent in the binge‐eating/purging subtype AN.

### 4.3 Clinical and Theoretical Implications

These findings support a shift away from relying solely on BMI‐based classification for determining severity in AN. While BMI may indicate the extent of dietary restriction, it inadequately captures psychiatric and functional aspects of severity.

In contrast, OWS and DT represent clinically relevant cognitive‐affective features. OWS appears to be a valuable first‐line indicator for identifying patients at risk of psychiatric burden and functional impairment. DT, though less prevalent, captures patients with more severe psychopathology. Their combination (OWS‐DT) demonstrated superior discriminative capacity across psychiatric, psychological, and functional domains, underscoring its utility as a dimensional severity marker.

Clinically, this two‐step screening approach is feasible: two items from the EDE‐Q (score ≥ 4) can identify patients OWS, and additional EDI‐2 items can assess DT. This stepwise approach aligns with the distribution patterns observed in our data and may help tailor interventions more precisely and better monitor illness trajectories. Theoretically, our findings emphasize the central role of cognitive‐affective features in AN severity beyond physical markers and call for greater integration of these dimensions in diagnostic systems such as the future DSM‐6.

### 4.4 Limitations and Perspectives

The current study has several limitations. Firstly, OWS and DT were analyzed as binary variables using previously validated cut‐offs (≥ 4 on either item for OWS and ≥ 14 on the total 7‐item score for DT). While these thresholds are commonly used and allow for comparability with prior research, they may not be optimal for all clinical settings and could lead to over‐or under‐estimations of prevalence. Moreover, applying fixed cut‐offs to continuous severity scales inherently limits the ability to capture the full range of symptom expression.

Secondly, since the sample consisted solely of treatment‐seeking patients who were predominantly female, it limits generalization to non‐treatment‐seeking individuals and to males. No data on race or ethnicity were collected, in accordance with French law, which limits the ability to assess potential confounding effects related to these demographic factors. Thirdly, the sample included only outpatients, potentially excluding individuals with more severe physiological consequences requiring higher levels of care. BMI may play a more clinically informative role in more acute treatment settings. Fourth, several psychological measures relied on self‐report, which may introduce reporting bias due to shame, low insight, denial, or attempts to elicit compassion. These factors may be particularly relevant in the subgroup with neither OWS nor DT, potentially obscuring symptom severity. Lastly, while the overall sample was sizable, subsamples for some comparisons, for example, by age (≤ 18), sex, or type of AN, were limited, preventing formal stratified analyses. For instance, although exploratory trends suggested differences in severity profiles between restricting and binge‐eating/purging subtypes, these findings require confirmation in larger, more balanced samples.

In addition, the study's cross‐sectional design precludes causal inference or evaluation of prognostic value. Future research should examine the predictive validity of OWS and DT through longitudinal studies, in terms of clinical outcomes, treatment response, or illness trajectory. Previous research has shown that DSM‐5 classification does not predict remission or recovery (Dalle Grave et al. 2018; Dang et al. 2025), reinforcing the need for alternative severity models. Future studies could benefit from treating OWS and DT as continuous constructs and exploring heterogeneity through methods such as latent class analysis, which may provide more nuanced insights into symptom profiles, particularly across different AN sub‐types.

Although no significant differences were found in calorimetry or blood tests with alternative criteria, this may be due to the limited statistical power and the lack of more sensitive and targeted biomarkers of somatic severity. Future studies should consider more targeted biological measures to better capture the somatic components of illness severity.

## 5 Conclusion

In conclusion, this study confirms the limited clinical utility of DSM‐5 severity categories based on BMI in assessing the overall severity of AN. Both OWS and DT independently captured important dimensions of severity, including comorbidities, psychopathology, and functional impairment. Their combined use (OWS‐DT) provided the greatest discriminative power, supporting its integration into clinical practice as a stepwise and easily applicable severity assessment tool. These findings highlight the need for a multidimensional approach to severity in AN that extends beyond weight‐based criteria.

## Author Contributions

**Esther Henriet:** writing – original draft, conceptualization, writing – review and editing, methodology, visualization. **Joanna Norton:** conceptualization, formal analysis, writing – original draft, writing – review and editing, visualization, methodology, validation. **Maude Seneque:** investigation, data curation, writing – review and editing. **Laurent Maimoun:** investigation, data curation, writing – review and editing. **Philippe Courtet:** project administration, resources, supervision, writing – review and editing. **Sébastien Guillaume:** conceptualization, methodology, project administration, resources, supervision, writing – review and editing.

## Conflicts of Interest

The authors declare no conflicts of interest.

## Supporting information


**Table S1:** History and psychopathology of anorexia nervosa according to severity classification, effect sizes with partial eta‐square coefficients (*η* ^2^) and 95% Confidence Intervals.

**Table S2:** Prevalence of psychiatric comorbidities across anorexia nervosa severity classifications, effect sizes with Cramer's V coefficients and 95% Confidence Intervals.

**Table S3:** Biological and metabolic markers according to severity classification, effect sizes with Cramer's V coefficients and 95% Confidence Intervals.

**Table S4:** Functional impact according to severity classification, effect sizes with partial eta‐square coefficients (*η* ^2^) and 95% Confidence Intervals.

**Figure S1:** Flow chart.

## Data Availability

The data that support the findings of this study are available from the corresponding author upon reasonable request.
