# Supplementary material for: Assessing Severity in Anorexia Nervosa Using Alternative Criteria to the DSM‐5 in a Cross‐Sectional Study
Source: Int J Eat Disord. 2025 Sep 10;58(12):2317–30. doi: 10.1002/eat.24542 (PMC12703215; doi:10.1002/eat.24542)
Supplement: Supplementary file 1 — Table S1: History and psychopathology of anorexia nervosa according to severity classification, effect sizes with partial eta‐square coefficients (η 2) and 95% Confidence Intervals. [file EAT-58-2317-s005.docx]

Supplementary Table 1. History and psychopathology of anorexia nervosa according to severity classification (N=312)^1^, effect sizes with partial eta-square coefficients (η²) and 95% Confidence Intervals

|  | DSM-5 severity |  | OWS |  | DT |  | OWS-DT |  |
| --- | --- | --- | --- | --- | --- | --- | --- | --- |
|  | η² (95%CI) |  | η² (95%CI) |  | η² (95%CI) |  | η² (95%CI) |  |
|  | p-value |  | p-value |  | p-value |  | p-value |  |
| Type of AN (Cramer’s V) | **0.26 (0.16-0.37)** |  | 0.18 (0.08-0.28) |  | 0.18 (0.06-0.28) |  | **0.22 (0.11-0.32)** |  |
|  | 0.0004 |  | 0.001 |  | 0.002 |  | 0.001 |  |
|  |  |  |  |  |  |  |  |  |
| Onset of AN | 0.002 (0.001-0.03) |  | 0.0009 (0.00-0.02) |  | 0.0009 (0.00-0.03) |  | 0.002 (0.00-0.04) |  |
|  | 0.94 |  | 0.64 |  | 0.64 |  | 0.82 |  |
|  |  |  |  |  |  |  |  |  |
| Illness duration | 0.02 (0.002-0.09) |  | 0.002 (0.00-0.03) |  | 0.007 (0.00-0.04) |  | 0.009 (0.001-0.05) |  |
|  | 0.2 |  | 0.5 |  | 0.22 |  | 0.32 |  |
|  |  |  |  |  |  |  |  |  |
| BMI at assessment | - |  | 0.02 (0.003-0.06) |  | 0.04 (0.008-0.09) |  | 0.05 (0.01-0.10) |  |
|  |  |  | 0.007 |  | 0.0009 |  | 0.0009 |  |
|  |  |  |  |  |  |  |  |  |
| Lowest BMI | **0.23 (0.16-0.34)** |  | 0.004 (0.00-0.03) |  | 0.004 (0.00-0.04) |  | 0.006 (0.00-0.04) |  |
|  | 0.0004 |  | 0.37 |  | 0.37 |  | 0.39 |  |
|  |  |  |  |  |  |  |  |  |
| 6-month change in BMI^2^ | **0.12 (0.06-0.21)** |  | 0.003 (0.00-0.03) |  | 0.0001 (0.00-0.02) |  | 0.001 (0.00-0.03) |  |
|  | 0.0004 |  | 0.82 |  | 0.87 |  | 0.87 |  |
| EDE-Q scores: |  |  |  |  |  |  |  |  |
| Restraint | 0.002 (0.00-0.03) |  | **0.16 (0.09-0.25)** |  | **0.33 (0.24-0.41)** |  | **0.35 (0.27-0.43)** |  |
|  | 0.92 |  | 0.0001 |  | 0.0001 |  | 0.0001 |  |
|  |  |  |  |  |  |  |  |  |
| Eating concern | 0.004 (0.009-0.04) |  | **0.25 (0.17-0.34)** |  | **0.31 (0.23-0.39)** |  | **0.40 (0.32-0.50)** |  |
|  | 0.72 |  | 0.0001 |  | 0.0001 |  | 0.0001 |  |
|  |  |  |  |  |  |  |  |  |
| Weight concern | 0.014 (0.002-0.06) |  | **0.31 (0.22-0.41)** |  | **0.41 (0.32-0.48)** |  | **0.51 (0.43-0.60)** |  |
|  | 0.22 |  | 0.0001 |  | 0.0001 |  | 0.0001 |  |
|  |  |  |  |  |  |  |  |  |
| Shape concern | 0.02 (0.003-0.07) |  | **0.35 (0.25-0.46)** |  | **0.36 (0.30-0.44)** |  | **0.52 (0.43-0.61)** |  |
|  | 0.13 |  | 0.0001 |  | 0.0001 |  | 0.0001 |  |
| EDI-2 scores: |  |  |  |  |  |  |  |  |
| Drive for thinness | | 0.03 (0.008-0.09) |  | 0.22 (0.14-0.31) |  | - |  | - |
|  | | 0.02 |  | 0.0002 |  |  |  |  |
|  | |  |  |  |  |  |  |  |
| Bulimia | | **0.06 (0.02-0.11)** |  | **0.07 (0.04-0.12)** |  | **0.13 (0.06-0.21)** |  | **0.15 (0.08-0.24)** |
|  | | 0.0004 |  | 0.0001 |  | 0.0001 |  | 0.0001 |
|  | |  |  |  |  |  |  |  |
| Body satisfaction | | 0.04 (0.02-0.10) |  | **0.14 (0.08-0.22)** |  | **0.32 (0.23-0.43)** |  | **0.34 (0.26-0.43)** |
|  | | 0.005 |  | 0.0001 |  | 0.0001 |  | 0.0001 |
|  | |  |  |  |  |  |  |  |
| Ineffectiveness | | 0.003 (0.00-0.03) |  | **0.15 (0.09-0.23)** |  | **0.20 (0.12-0.29)** |  | **0.25 (0.18-0.34)** |
|  | | 0.82 |  | 0.0001 |  | 0.0001 |  | 0.0001 |
|  | |  |  |  |  |  |  |  |
| Perfectionism | | 0.02 (0.003-0.06) |  | **0.06 (0.02-0.11)** |  | **0.09 (0.04-0.16)** |  | **0.10 (0.05-0.18)** |
|  | | 0.19 |  | 0.0001 |  | 0.0001 |  | 0.0001 |
|  | |  |  |  |  |  |  |  |
| Interpersonal distrust | | 0.002 (0.001-0.03) |  | 0.03 (0.004-0.06) |  | 0.01 (0.00-0.05) |  | 0.03 (0.009-0.08) |
|  | | 0.91 |  | 0.0001 |  | 0.07 |  | 0.008 |
|  | |  |  |  |  |  |  |  |
| Interoceptive | | 0.01 (0.001-0.05) |  | **0.17 (0.10-0.24)** |  | **0.24 (0.16-0.32)** |  | **0.29 (0.22-0.38)** |
| awareness | | 0.27 |  | 0.0002 |  | 0.0002 |  | 0.0001 |
|  | |  |  |  |  |  |  |  |
| Maturity fears | | 0.007(0.001-0.05) |  | 0.04 (0.01-0.09) |  | **0.07 (0.02-0.14)** |  | **0.08 (0.03-0.15)** |
|  | | 0.5 |  | 0.0001 |  | 0.0001 |  | 0.0001 |
|  | |  |  |  |  |  |  |  |
| Asceticism | | 0.01 (0.002-0.05) |  | **0.12 (0.06-0.18)** |  | **0.23 (0.15-0.31)** |  | **0.25 (0.18-0.33)** |
|  | | 0.33 |  | 0.0001 |  | 0.0001 |  | 0.0001 |
|  | |  |  |  |  |  |  |  |
| Impulse regulation | | 0.005 (0.001-0.04) |  | **0.11 (0.06-0.18)** |  | **0.12 (0.06-0.20)** |  | **0.17 (0.10-0.26)** |
|  | | 0.65 |  | 0.0001 |  | 0.0001 |  | 0.0001 |
|  | |  |  |  |  |  |  |  |
| Social insecurity | | 0.006 (0.001-0.04) |  | **0.10 (0.04-0.17)** |  | **0.10 (0.04-0.17)** |  | **0.15 (0.09-0.24)** |
|  | 0.61 |  | 0.0001 |  | 0.0001 |  | 0.0001 |  |

Note: AN= anorexia nervosa, OWS= overvaluation of weight and shape, DT= drive for thinness, BMI= body mass index expressed in kg/m^2^, EDE-Q= Eating Disorder Examination Questionnaire, EDI-2= Eating Disorder Inventory

SD= standard deviation, η^2^= partial eta-squared coefficients effect size for continuous variables

^1^missing values: for EDE-Q and EDI-2 (≤1.5%); BMI at assessment (6%); duration of illness, lowest BMI and change in BMI (18-20%)

^2^Difference between BMI at 6 months prior to assessment and BMI at assessment

In bold: medium to large effect sizes based on Cramer’s V with the following thresholds:

for DSM-5 severity level (3 df): [0.06 - 0.17[: small, [0.17 - 0.29[: medium, [0.29 +: large ;

for OWS and DT (1 df): [0.10 - 0.30[: small, [0.30 - 0.50[: medium, [ 0.50 +: large ;

for OWS-DT (2 df): [0.07 - 0.21[: small, [0.21 - 0.35[: medium, [ 0.35 +: large

In bold: medium to large effect sizes for continuous variables, based on partial eta-square (η2) with the following thresholds:

0.01≤η^2^<0.06: small;

0.06≤η^2^<0.14: medium;

η^2^≥: 0.14: large
