# Supplementary material for: Assessing Severity in Anorexia Nervosa Using Alternative Criteria to the DSM‐5 in a Cross‐Sectional Study
Source: Int J Eat Disord. 2025 Sep 10;58(12):2317–30. doi: 10.1002/eat.24542 (PMC12703215; doi:10.1002/eat.24542)
Supplement: Supplementary file 2 — Table S2: Prevalence of psychiatric comorbidities across anorexia nervosa severity classifications, effect sizes with Cramer's V coefficients and 95% Confidence Intervals. [file EAT-58-2317-s001.docx]

Supplementary Table 2. Prevalence of psychiatric comorbidities across anorexia nervosa severity classifications (N=312) ^1^, effect sizes with Cramer’s V coefficients and 95% Confidence Intervals

|  | DSM-5 severity |  | OWS |  | DT |  | OWS-DT |
| --- | --- | --- | --- | --- | --- | --- | --- |
|  | Cramer’s V (95%CI)  p-value |  | Cramer’s V (95%CI)  p-value |  | Cramer’s V (95%CI)  p-value |  | Cramer’s V (95%CI)  p-value |
| Past hospitalisation in psychiatry | 0.11 (0.05-0.24) |  | 0.10 (0.01-0.21) |  | 0.17 (0.06-0.28) |  | 0.16 (0.07-0.28) |
|  | 0.28 |  | 0.11 |  | 0.02 |  | 0.04 |
|  |  |  |  |  |  |  |  |
| Suicide Attempt | **0.21 (0.13-0.30)** |  | 0.13 (0.03-0.22) |  | 0.20 (0.09-0.31) |  | 0.20 (0.10-0.31) |
|  | 0.005 |  | 0.02 |  | 0.002 |  | 0.004 |
|  |  |  |  |  |  |  |  |
| Current MDD | 0.07 (0.03-0.20) |  | 0.18 (0.07-0.28) |  | 0.23 (0.11-0.35) |  | **0.25 (0.15-0.36)** |
|  | 0.7 |  | 0.003 |  | 0.0002 |  | 0.0002 |
|  |  |  |  |  |  |  |  |
| Past MDD | **0.17 (0.09-0.30)** |  | 0.21 (0.09-0.32) |  | 0.09 (0.005-0.20) |  | **0.21 (0.10-0.33)** |
|  | 0.04 |  | 0.002 |  | 0.14 |  | 0.004 |
|  |  |  |  |  |  |  |  |
| Anxiety Disorders | 0.08 (0.04-0.22) |  | 0.28 (0.16-0.39) |  | 0.16 (0.05-0.28) |  | **0.29 (0.20-0.40)** |
|  | 0.62 |  | 0.0002 |  | 0.009 |  | 0.0002 |
|  |  |  |  |  |  |  |  |
| OCD | 0.09 (0.04-0.22) |  | 0.13 (0.03-0.22) |  | 0.10 (0.01-0.21) |  | 0.14 (0.04-0.25) |
|  | 0.55 |  | 0.12 |  | 0.12 |  | 0.12 |

Note: OWS= overvaluation of weight and shape, DT= drive for thinness, MDD= major depressive disorder, OCD= obsessive compulsive disorder

V= Cramer’s V effect size for categorial variables

^1^missing values: ≤3% for current MDD, past MDD, anxiety disorders, OCD

In bold: medium to large effect sizes based on Cramer’s V with the following thresholds:

for DSM-5 severity level (3 df): [0.06 - 0.17[: small, [0.17 - 0.29[: medium, [0.29 +: large ;

for OWS and DT (1 df): [0.10 - 0.30[: small, [0.30 - 0.50[: medium, [ 0.50 +: large ;

for OWS-DT (2 df): [0.07 - 0.21[: small, [0.21 - 0.35[: medium, [ 0.35 +: large
