# Supplementary material for: Assessing Severity in Anorexia Nervosa Using Alternative Criteria to the DSM‐5 in a Cross‐Sectional Study
Source: Int J Eat Disord. 2025 Sep 10;58(12):2317–30. doi: 10.1002/eat.24542 (PMC12703215; doi:10.1002/eat.24542)
Supplement: Supplementary file 3 — Table S3: Biological and metabolic markers according to severity classification, effect sizes with Cramer's V coefficients and 95% Confidence Intervals. [file EAT-58-2317-s003.docx]

Supplementary Table 3. Biological and metabolic markers according to severity classification (N=312)^1^, effect sizes with Cramer’s V coefficients and 95% Confidence Intervals

|  | DSM-5 severity |  | OWS |  | DT |  | OWS-DT |
| --- | --- | --- | --- | --- | --- | --- | --- |
|  | Cramer’s V (95%CI)  p-value |  | Cramer’s V (95%CI)  p-value |  | Cramer’s V (95%CI)  p-value |  | Cramer’s V (95%CI)  p-value |
| Resting energy | **0.35 (0.24-0.45)** |  | 0.09 (0.006-0.21) |  | 0.008 (0.002-0.13) |  | 0.07 (0.02-0.18) |
| expenditure (terciles) | <0.0001 |  | 0.24 |  | 0.89 |  | 0.67 |
|  |  |  |  |  |  |  |  |
| Hepatic assessment | 0.10 (0.04-0.23) |  | 0.03 (0.002-0.14) |  | 0.10 (0.009-0.20) |  | 0.14 (0.05-0.24) |
|  | 0.56 |  | 0.66 |  | 0.18 |  | 0.18 |
|  |  |  |  |  |  |  |  |
| Deficiency in all 5 biological tests | 0.15 (0.07-0.26) |  | 0.05 (0.003-0.15) |  | 0.10 (0.01-0.25) |  | 0.13 (0.04-0.25) |
|  | 0.15 |  | 0.39 |  | 0.15 |  | 0.15 |
|  |  |  |  |  |  |  |  |
| Biol. deficiency and hepatic impairment | 0.08 (0.04-0.21) |  | 0.07 (0.004-0.16) |  | 0.14 (0.01-0.25) |  | 0.17 (0.06-0.28) |
|  | 0.62 |  | 0.35 |  | 0.06 |  | 0.06 |
|  |  |  |  |  |  |  |  |

Note: OWS= overvaluation of weight and shape, DT= drive for thinness, Resting energy expenditure (terciles)= deviation from normative values as percentages, T1&T2= first two terciles deviating from normative values, T3= lowest tercile as in most pronounced reduction in resting energy expenditure

V= Cramer’s V effect size for categorial variables

^1^missing values: ≤3% for resting energy expenditure and hepatic assessment; 15% for biological deficiency

In bold : medium to large effect sizes based on Cramer’s V with the following thresholds:

for DSM-5 severity level (3 df): [0.06 - 0.17[: small, [0.17 - 0.29[: medium, [0.29 +: large ;

for OWS and DT (1 df): [0.10 - 0.30[: small, [0.30 - 0.50[: medium, [ 0.50 +: large ;

for OWS-DT (2 df): [0.07 - 0.21[: small, [021 - 0.35[: medium, [ 0.35 +: large
