# Supplementary material for: Assessing Severity in Anorexia Nervosa Using Alternative Criteria to the DSM‐5 in a Cross‐Sectional Study
Source: Int J Eat Disord. 2025 Sep 10;58(12):2317–30. doi: 10.1002/eat.24542 (PMC12703215; doi:10.1002/eat.24542)
Supplement: Supplementary file 4 — Table S4: Functional impact according to severity classification, effect sizes with partial eta‐square coefficients (η 2) and 95% Confidence Intervals. [file EAT-58-2317-s002.docx]

Supplementary Table 4. Functional impact according to severity classification (N=163)^1^, effect sizes with partial eta-square coefficients (η²) and 95% Confidence Intervals

|  | DSM-5 severity |  | OWS |  | DT |  | OWS-DT |
| --- | --- | --- | --- | --- | --- | --- | --- |
|  | η² (95%CI)  p-value |  | η² (95%CI)  p-value |  | η² (95%CI)  p-value |  | η² (95%CI)  p-value |
| WSAS score | 0.03 (0.006-0.12) |  | **0.07 (0.01-0.18)** |  | **0.06 (0.01-0.14)** |  | **0.12 (0.04-0.23)** |
|  | 0.18 |  | 0.001 |  | 0.001 |  | 0.001 |
|  |  |  |  |  |  |  |  |
| EDQOL |  |  |  |  |  |  |  |
| Psychological | 0.03 (0.007-0.10) |  | **0.17 (0.08-0.29)** |  | **0.21 (0.12-0.31)** |  | **0.28 (0.19-0.41)** |
|  | 0.17 |  | 0.001 |  | 0.001 |  | 0.001 |
|  |  |  |  |  |  |  |  |
| Physiological | 0.02 (0.003-0.09) |  | 0.04 (0.003-0.12) |  | **0.08 (0.01-0.15)** |  | **0.08 (0.02-0.19)** |
|  | 0.42 |  | 0.01 |  | 0.002 |  | 0.002 |
|  |  |  |  |  |  |  |  |
| Financial | 0.0005 (0.00-0.08) |  | 0.02 (0.00-0.08) |  | 0.03 (0.00-0.11) |  | 0.04 (0.006-0.13) |
|  | 0.99 |  | 0.17 |  | 0.11 |  | 0.14 |
|  |  |  |  |  |  |  |  |
| Work | 0.01 (0.002-0.09) |  | 0.04 (0.003-0.10) |  | **0.07 (0.01-0.17)** |  | **0.08 (0.03-0.20)** |
|  | 0.68 |  | 0.01 |  | 0.004 |  | 0.004 |

Note: OWS= overvaluation of weight and shape, DT= drive for thinness, WSAS= Work and Social Adjustment Scale, EDQOL= Eating Disorders Quality of Life Questionnaire

SD= standard deviation, η^2^= partial eta-square (η^2^) effect size for continuous variables

^1^missing values for EDQOL: 9% for Work; 11% for Financial

In bold: medium to large effect sizes based on partial eta-square (η2) with the following thresholds:

0.01≤η2<0.06: small; 0.06≤η2<0.14: medium; η2≥: 0.14: large
